# The Impact of Diabetic Neuropathy on Balance and on the Risk of Falls in Patients with Type 2 Diabetes Mellitus: A Cross-Sectional Study

**DOI:** 10.1371/journal.pone.0154654

**Published:** 2016-04-27

**Authors:** Bogdan Timar, Romulus Timar, Laura Gaiță, Cristian Oancea, Codrina Levai, Diana Lungeanu

**Affiliations:** 1 Department of Functional Sciences,”Victor Babes” University of Medicine and Pharmacy, Timisoara, Romania; 2 2^nd^ Department of Internal Medicine,”Victor Babes” University of Medicine and Pharmacy, Timisoara, Romania; 3 Department of Cardiology,”Victor Babes” University of Medicine and Pharmacy, Timisoara, Romania; 4 Department of Infectious Diseases,”Victor Babes” University of Medicine and Pharmacy, Timisoara, Romania; 5 Legal Department,”Victor Babes” University of Medicine and Pharmacy, Timisoara, Romania; Sapienza, University of Rome, School of Medicine and Psycology, ITALY

## Abstract

**Introduction:**

Diabetic neuropathy (DN) is a prevalent complication of Type 2 Diabetes Mellitus (T2DM) with a major impact on the health of the affected patient. We hypothesized that mediated by the dysfunctionalities associated with DN’s three major components: sensitive (lack of motion associated sensory), motor (impairments in movement coordination) and autonomic (the presence of postural hypotension), the presence of DN may impair the balance in the affected patients. Our study’s main aim is to evaluate the possible association between the presence and severity of DN and both the balance impairment and the risk of falls in patients with T2DM.

**Material and Method:**

In this cross-sectional study we enrolled, according to a consecutive-case population-based setting 198 patients with T2DM. The presence and severity of DN was evaluated using the Michigan Neuropathy Screening Instrument, a tool which allows both diagnosing and severity staging of DN. The balance impairment and the risk of falls were evaluated using four validated and standardized tools: Berg Balance Scale (BBS), Timed-up and Go test (TUG), Single Leg Stand test (SLS) and Fall Efficacy Scale (FES-I).

**Results:**

The presence of DN was associated with significant decreases in the BBS score (40.5 vs. 43.7 points; p<0.001) and SLS time (9.3 vs. 10.3 seconds; p = 0.003) respectively increases in TUG time (8.9 vs. 7.6 seconds; p = 0.002) and FES-I score (38 vs. 33 points; p = 0.034). The MNSI score was reverse and significantly correlated with both BBS score (Spearman’s r = -0.479; p<0.001) and SLS time (Spearman’s r = -0.169; p = 0.017). In the multivariate regression model, we observed that patient’s age, DN severity and depression’s symptoms acted as independent, significant predictors for the risk of falls in patients with T2DM.

**Conclusions:**

The presence of DN in patients with DM is associated with impaired balance and with a consecutively increase in the risk of falls.

## Introduction

Diabetes mellitus (DM) is one of the largest global public health emergencies of the 21st century. Approximately 415 million adults have DM and by 2040 this number will rise to 642 million. [1] In Romania, there are 1.5–1.9 million diabetic patients, with a prevalence of 11.6%. [2]

It is estimated that 193 million people with diabetes are undiagnosed and are therefore more at risk of developing complications. [1] Consistently high blood glucose levels can affect the heart and blood vessels, eyes, kidneys and nerves. [3] Biochemical abnormalities are causing protein glycation and overproduction of reactive oxygen species, leading to vascular damage and responsive activation of tissue-specific growth/repair systems. [4]

Neuropathies are one of the most common complication of DM [5] with a prevalence of approximately 60%. [6] Patients with type 2 DM (T2DM) may present with this complication after only a few years of known poor glycemic control; sometimes, these patients already have neuropathy at the time of diagnosis.

Diabetic neuropathy (DN) severely decreases patients' quality of life [5] and the quality of diabetes self-management and, in consequence, is worsening the prognosis of other diabetes complications. Some patients cannot sense temperature and often burn themselves during daily activities. Lack of sensitivity, or loss of nociception can lead to foot ulceration and to unintentional but serious injuries that can become infected and lead to amputation. **[**7] Recent research brought to attention that cardiac autonomic neuropathy is independently associated with chronic kidney disease in patients with type 2 diabetes [8] and that diabetic neuropathy and albuminuria are significantly associated. [9] In addition, cardiac autonomic neuropathy is associated with increased cardiovascular morbidity and mortality. [10]

DN may compromise balance during daily activities. Patients with DN have a fivefold increased risk of falling [11] and the consequences include decline in mobility, activity avoidance, institutionalization and mortality. [12] This balance impairment in patients with DN is predominantly in the medial-lateral plane and is the greatest during stair descent. [11] Both physiological (strength and proprioception) and cognitive–behavioral factors (fear of falls) should be considered when treating diabetic patients with gait alterations. [13]

We hypothesized that the presence of DN may impair the balance of the affected patients mediated by the dysfunctionalities associated with all of its three major components: sensitive (lack of motion associated sensory), motor (impairments in movement coordination) and autonomic (the presence of postural hypotension). The main aim of our study is to evaluate the possible association between the presence and severity of DN and balance impairment and the risk of falls in patients with T2DM.

## Material and Method

### Study design and patients

In this cross-sectional, non-interventional study, we enrolled 198 patients previously diagnosed with Type 2 Diabetes, attending scheduled visits in the Center for Diabetes Treatment of the Emergency Hospital Timisoara, Romania. Patients participating in the study were enrolled according a population based, consecutive-case principle. The study design, protocol and informed consent form were reviewed and approved by the Ethics Committee of the Emergency Hospital Timisoara (no. 328/2014); all patients provided written informed consent prior any study procedure or activity.

At the time of the screening patients the following were considered exclusion criteria: the inability to provide informed consent, inability to provide accurate anamnestic medical history data, prior history of non-diabetic neuropathies, major cardiovascular events (according to Hicks 2014 criteria) 3 months prior to screening, or any other condition which, in the investigators’ opinion, could lead to biases in the study results.

The enrolled patients had a median age of 61 years, a median duration of diabetes of 7 years, 44.9% of them (n = 89) being males.

### Diabetic neuropathy assessment

The Michigan Neuropathy Screening Instrument (MNSI) was used to diagnose DN. MNSI is a validated score instrument for DN, being widely used for the diagnosis and quantification of diabetic distal symmetrical peripheral neuropathy. The MNSI consists of two distinct parts: a 15-item self-administered questionnaire that is scored by summing abnormal answers provided by the patient and a lower extremity examination performed by the healthcare professional, including inspection, evaluation of ankle reflexes and of vibration perception at great toe, in which the abnormal findings are scored. The MNSI has both higher sensitivity and specificity than individual DN tests separately; a self-administered questionnaire scoring 7 or above, or a clinical examination score of 2.5 or above provided a positive diagnosis of DN, which was considered more severe with the increase of the MNSI score. [14]

The presence of cardiac autonomic neuropathy, which is a frequent condition associated with diabetic polyneuropathies, was investigated through the measurement of changes in postural blood pressure. A positive diagnosis for postural hypotension was represented by a drop in systolic blood pressure of 20 mmHg or diastolic blood pressure of 10 mmHg within three minutes after changing the body position from supine to standing.

### Balance assessment

The presence and severity of balance impairment were evaluated using the following four components: the Berg Balance Scale (BBS), Single leg stand test (SLS), Timed-up and go test (TUG) and using the Fall Efficacy Scale—International (FES-I) questionnaire.

The gold standard test for static and dynamic balance abilities is the BSS test. It is a valid instrument used for the evaluation of the interventions’ effectiveness and for the quantitative description of function in clinical practice and research. It consists of a 14 item scale for simple balance tasks (postural changes and positions, transfers and simple object retrieval maneuvers). The ability of performing each task is given a score from 0 (unable) to 4 (independent) and the final result is the sum of all scores; the lower the score the more severe the balance impairment. [15]

The quantification of functional mobility is achieved with TUG test, a reliable and valid test, which may also be useful in following clinical change over time. The TUG test is quick, it doesn’t require any training or special equipment, and can easily be included as part of the routine medical examination. The patient is observed and timed in the time interval he requires to rise from an arm chair, walk 3 meters, turn, walk back, and sit down again. Static and dynamic balance impairment is translated in a longer duration needed to perform the test’s tasks. [16]

SLS tests static balance and records the time a participant is able to stand on one leg unassisted. Participants were encouraged to rest as needed throughout the assessment session. The TUG and SLS tests were performed thrice with pause intervals between repetition sets and the best value was used. Static balance impairment was revealed by a lower SLS time.

FES-I is a widely accepted tool for measuring falling likeliness. It is a self-report questionnaire which assesses the concern about falling during a range of routine activities; it contains 16 items, each having a four-point scale (ranging from 1 that means not concerned at all to 4 very concerned). The higher the FES-I score, the higher the probability of falls in the examined patient, based on their personal perception of balance impairment. [17]

### Clinical, anthropometric and laboratory data

Data regarding patient age, diabetes history—including the history of diabetes treatment, smoking history and body mass index were collected from the patients’ medical records. The HbA1c level was measured using a NGSP-standardized and DCCT-compliant immune-turbidimetric assay (Roche), having an inter-measurement coefficient of variation of 1.64% according to manufacturer’s specifications. The presence and severity of chronic kidney disease was diagnosed according to Kidney Disease: Improving Global Outcomes 2012 guidelines. Retinopathy diagnosis was established after performing a funduscopic examination by the same trained ophthalmologist, specialized in the diagnosis and treatment of diabetic eye complications.

To evaluate the presence and the severity of depression we used the Patient’s Health Questionnaire– 9 (PHQ-9). The nine criteria on which the diagnosis of DSM-IV depressive disorders is based are comprised within the nine items the PHQ-9 test consists of. The PHO-9 can establish, with the 9 items, both depressive distress as well as the grade of the depressive symptom severity, being a dual-purpose instrument. A higher PHQ-9 score is associated with more severe depression; based on this score, the depression’s severity may be scaled as follows, in three degrees: minimal or mild (PHQ-9 score < 10), moderate (PHQ-9 score 10–19) and severe (PHQ score >19). [18]

### Statistical analysis

Data were collected and analyzed using SPSS v17 statistical software package (SPSS Inc, Chicago, IL, USA) and are presented as average ± standard deviation (numerical variables with Gaussian distribution), median and [interquartile range] (numerical variables with non-parametric distributions) respectively percentage from the sub-group total and (number of individuals). To assess the significance of the differences between groups, the Student t-test (means, Gaussian populations), Mann-Whitney U (medians, non-parametric populations) and Chi-square (proportions) were used. Continuous variables distributions were tested for normality using Shapiro-Wilk test, and for equality of variances using Levene’s test.

To evaluate the strength of the association between two continuous variables we used Spearman’s correlation coefficient; the statistical significance of the correlation was evaluated using t-distribution test. The individual impact of several confounding factors on the variance of a continuous variable was assessed by building multivariate regression models. The predictors, in the final regression equations, were accepted according to a repeated backward-stepwise algorithm (inclusion criteria p<0.05, exclusion criteria p>0.10) in order to obtain the most appropriate theoretical model to fit the collected data. The quality of the model was described using the accuracy of the prediction by adjusted R2 (multivariate linear regression).

In this study, a p-value of 0.05 was considered the threshold for statistical significance.

### Data availability statement

All relevant raw data used in this study can be downloaded from https://dx.doi.org/10.6084/m9.figshare.3176152

## Results

### Neuropathy and neuropathy related associations

In our study cohort, the prevalence of diabetic neuropathy, as diagnosed according to the MNSI criteria was 28.8% (57 cases). The presence of overt neuropathy was associated with an increased age (64.5 vs. 59 years; p = 0.001), BMI (31.9 vs. 29.9 kg/m2; p = 0.030), depression severity—assessed using the PHQ-9 score (12.2 vs. 7.2 points; p = 0.011). Patients with positive diagnosis of depression had a higher prevalence of other diabetes complications: chronic kidney disease (56.1% vs. 14.2%; p<0.001) and retinopathy (54.4% vs. 22.0%). The differences in the other studied parameters had no significant differences between the groups: diabetes duration, HbA1c, prevalence of smoking and hypertension (Table 1).

**Table 1:** Patient’s characteristics, stratified according to the presence of diabetic neuropathy.

	Without overt neuropathy	Overt neuropathy present	p
Male gender ^a^	64 (45.4%)	25 (43.9%)	0.845
Age (years) ^b^	59 [12]	64.5 [10.5]	0.001*
Diabetes duration (years) ^b^	7 [9]	7 [9]	0.867
HbA1c (%) ^c^	8.0 ± 1.8	8.6 ± 1.6	0.620
BMI (kg/m^2^) ^c^	29.9 ± 4.3	31.9 ± 3.9	0.030*
PHQ-9 score (points) ^c^	7.2 ± 5.3	12.2 ± 7.9	0.011*
Smokers ^a^	50 (35.5%)	15 (26.3%)	0.215
Hypertensive patients ^a^	117 (83.0%)	45 (78.9%)	0.505
Chronic kidney disease ^a^	20 (14.2%)	32 (56.1%)	<0.001*
Retinopathy ^a^	31 (22.0%)	31 (54.4%)	<0.001*

* *Differences are statistically significant at α<0.05 threshold*

*^a^ Dichotomous variables. Results are presented as number of individuals and (percentage from the sub-group). p value was calculated using chi-square test*.

*^b^ Numerical variables with non-parametric distribution. Results are presented as median and [interquartile range]. p value was calculated using Mann-Whitney U test*.

*^c^ Numerical variables with Gaussian distribution. Results are presented as average ± standard deviation. p value was calculated using unpaired t-student test*.

HbA1c –Hemoglobin A1c

BMI—Body mass index

PHQ-9 –Patient’s health questionnaire-9

### Neuropathy impact on balance parameters

In our study, the presence of overt neuropathy, diagnosed as presented in the Method section—based on the MNSI score, was associated with a decreased BBS (40.5 vs. 43.7 points; p<0.001), SLS (9.3 vs. 10.2 s; p = 0.003), respectively with an increased TUG (8.9 vs. 7.6 s; p = 0.002) and FES-I questionnaire score (38 vs. 33 points; p = 0.034). These results are pointing to a significant, negative impact of DN on the patient’s balance parameters (Table 2), leading thus, indirectly, to an increased risk of falls. According to our results, based of the interpretation of the BBS, in the sub-group of patients with DN (n = 57), 52.6% of the patients (n = 30) had balance impairment in contrast to only 32.6% of the patients without DN (p<0.001).

**Table 2:** Balance parameters in patients with vs. without diabetic neuropathy.

	Without overt neuropathy	Overt neuropathy present	p
BBS (points) ^a^	43.7 ± 5.0	40.5 ± 5.3	<0.001*
TUG (s) ^b^	7.6 [3.1]	8.9 [3.0]	0.002*
SLS (s) ^a^	10.3 ± 2.0	9.3 ± 1.8	0.003*
FES—I (points) ^b^	33 [14.0]	38 [12]	0.034*

* *Differences are statistically significant at α<0.05 threshold*

*^a^ Numerical variables with Gaussian distribution. Results are presented as average ± standard deviation. p value was calculated using unpaired t-student test*.

*^b^ Numerical variables with non-parametric distribution. Results are presented as median and [interquartile range]. p value was calculated using Mann-Whitney U test*.

BBS—Berg Balance Scale

TUG—Timed up and go

SLS—Single leg stand

FES-I—Falls Efficacy Scale—International

**Fig 1:**
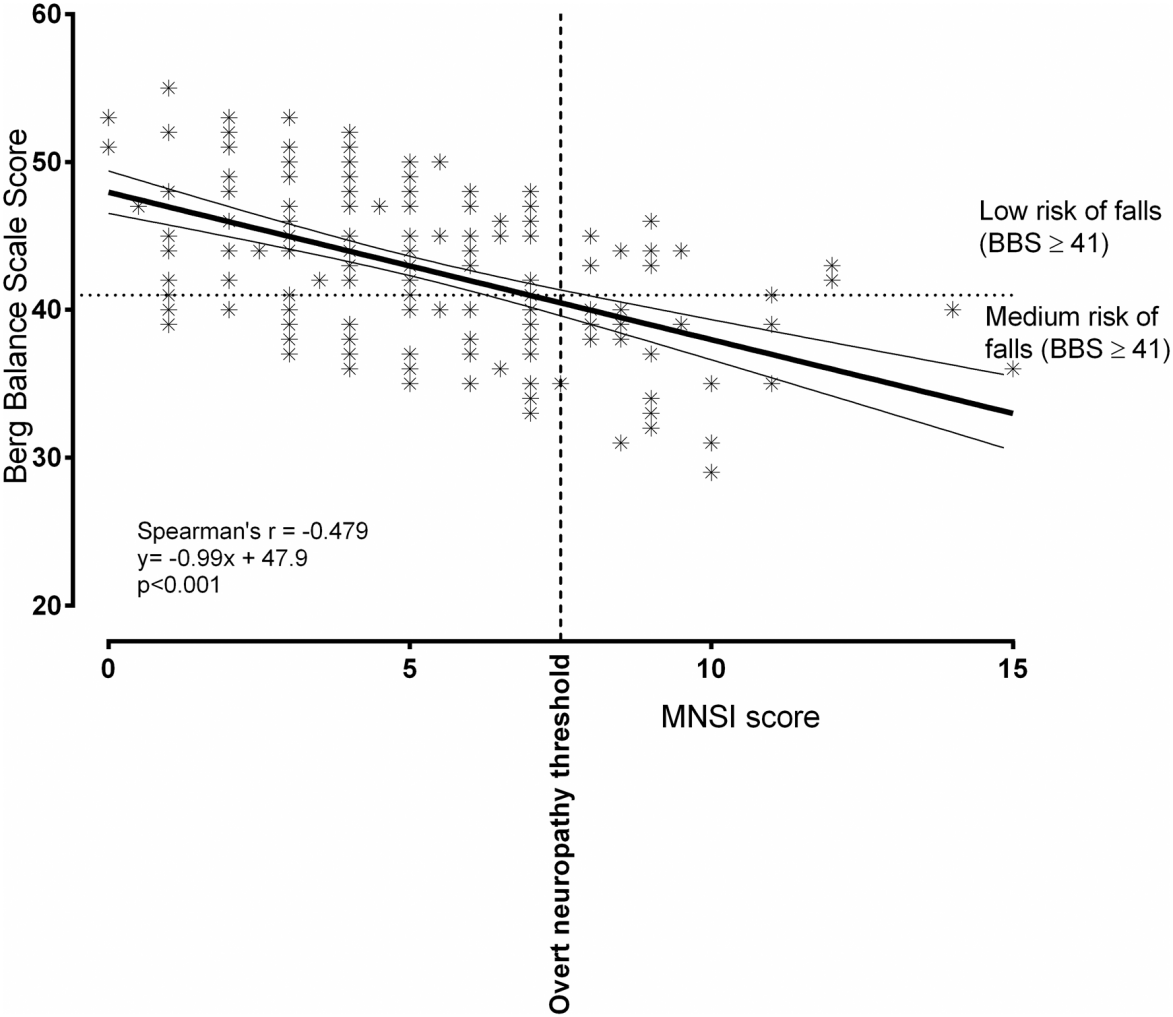
The correlation between MNSI and BBS score. MNSI—Michigan Neuropathy Screening Instrument; BBS—Berg Balance Scale.

The MNSI score, which also describes the severity of DN was significantly and reverse correlated with BBS (Spearman’s r = -0.479; p<0.001; Fig 1) and SLS time (r = -0.169; p = 0.017), demonstrating that not only the presence of neuropathy, but also its severity has a negative impact on the balance parameters (Table 3). The correlations analyzed between the MNSI score and TUG and FES-I questionnaire score were weak and positive, however the relationship proved to be statistically non-significant.

**Table 3:** Correlations between neuropathy’s severity and balance parameters.

	MSNI score (points)	p
BBS (points)	-0.479	<0.001*
TUG (s)	0.122	0.088
SLS (s)	-0.169	0.017*
FES—I (points)	0.116	0.104

* *Correlations are statistically significant at α<0.05 threshold*

MNSI—Michigan Neuropathy Screening Instrument

BBS—Berg Balance Scale

TUG—Timed up and go

SLS—Single leg stand

FES-I—Falls Efficacy Scale—International

The presence of orthostatic hypotension, a possible marker of cardiac autonomic neuropathy, was associated with a decreased SLS time (9.3 vs. 10.2 seconds; p = 0.028) and with an increased score in the subjective, FES-I questionnaire (37 vs. 32 points; p = 0.048). The other two studied components regarding the possible balance impairment (BBS and TUG) had no significant differences between the group of patients with vs. without orthostatic hypotension (Table 4).

**Table 4:** Balance parameters in patients with vs. without orthostatic hypotension.

	Orthostatic hypotension absent	Orthostatic hypotension present	p
BBS (points)	42.8 ± 5.3	42.7 ± 5.4	0.902
TUG (s)	7.8 [2.9]	8.7 [3.2]	0.054
SLS (s)	10.2 ± 2.0	9.3 ± 1.9	0.028*
FES—I (points)	32 [13]	37 [16]	0.048*

* *Differences are statistically significant at α<0.05 threshold*

*^a^ Numerical variables with Gaussian distribution. Results are presented as average ± standard deviation. p value was calculated using unpaired t-student test*.

*^b^ Numerical variables with non-parametric distribution. Results are presented as median and [interquartile range]. p value was calculated using Mann-Whitney U test*.

BBS—Berg Balance Scale

TUG—Timed up and go

SLS—Single leg stand

FES-I—Falls Efficacy Scale—International

### Predictors for balance impairment in patients with T2DM

Since the presence of DN is one complication of DM and there are a series of other co-factors involved in balance impairment in these patients we aimed to evaluate which other factors are possible predictors and if considering these factors, the severity of DN keeps its influence on the balance parameters independently. For this, using the other collected parameters (added in the regression model respecting a stepwise backward algorithm) we built a multivariate regression model having as outcome the BBS score. The final model, which proved to describe the best the studied relationship contained the following parameters: MNSI score, Patient’s age, HbA1c value, depression (PHQ-9 score).

We observed that a significant impact on the BBS had the severity of neuropathy, patient’s age and the severity of depression. These predictors also proved to act as independent factors in the development of balance impairments and not only as co-founding factors. According to the multivariate regression model we observed that for an increase with one standard deviation in the MNSI score, the BBS score decreased with 0.622 standard deviations (p<0.001). In the same time, the inferring of the regression equation suggested that for an increase with one standard deviation in patient’s age we might expect a decrease with 0.1 standard deviations in BBS score (p = 0.015) while for an increase with one standard deviation in depression score (PHQ-9) we might expect a decrease in the BBS score with 0.183 standard deviations (p = 0.021). The detailed results of the regression analysis are presented in Table 5.

**Table 5:** Multivariate regression analysis results (dependent variable: BBS score).

	B	Exp (β)	p
MNSI score (points)	-1.208	-0.622	<0.001
Age (years)	-0.051	-0.100	0.021
PHQ-9 (points)	-0.149	-0.183	0.015

## Discussion

### Findings and interpretation

The results of our study are pointing to a significant association between DN and balance impairment and indirectly with the risk of falls in patients with T2DM. Not only that the presence of DN leads to impairments of the balance, but also its severity is strongly correlated with the balance impairment’s degree. In our study cohort, similar to what is already described in the literature regarding patients with T2DM [19] the presence of DN was frequently associated with the development of cardiac autonomic neuropathy. This association, most probably mediated by the consequences of cardiac autonomic neuropathy (e.g. postural hypotension) is further emphasizing the balance impairment and is increasing the risk of falls in these patients.

It is known that the development of DN is also associated, besides an inadequate long-term glycemic control, with other several factors which may also have an impact on the development of balance impairment (e.g. patient’s age, gender, the presence of other diabetes chronic complications or co-morbidities) raising thus the question of whether or not DN is just a confounding factor associated with these predictors [12]. The multivariate regression model built, in which the predictors were included both according to the stepwise criterion but also according to the information’s entropy law criterion postulated by Akaike [20], demonstrated that the severity of DN acts also independently predicting the balance impairment and not only as a co-factor along the other significant predictors.

### Strengths and weaknesses of the study

The strengths of the study are emphasized by the patients’ consecutive-enrollment principle which provided a heterogeneous study cohort having the baseline characteristics matching the characteristics of the T2DM general population, containing both patients with overt DN and patients without any signs of DN. Also, the age and diabetes duration profile of our study group was heterogeneous, in contrast to similar studies [21], containing not only patients with advanced ages and longer diabetes duration. The sample size is another strong-point of our study design, providing enough statistical power to reject the null hypothesis when this is not valid in the population and thus allowing the inference of the results obtained in the sample for the population of interest.

The main weak point is the cross-sectional design of the study and its relation to the association between DN and inadequate glycemic control. It is known that the development of DN as well as other T2DM complication is a long-term process, and the cross-sectional evaluation of the glycemic control (in our study the cross sectional value of HbA1c –an indicator which may reflect the quality of the glycemic control for no longer than 3 months) may not always be associated with the development of T2DM complications. However, this weak point of the study is not interfering with the study’s main aim, which is to evaluate the relationship between the already established DN and balance impairment.

### Relevance of the findings

Our findings are demonstrating that balance impairment and the risk of falls, many times underestimated and under-screened, are a frequent condition in patients having DN. More, the impairments observed are more severe as the DN symptomatology increases. Since DN is a frequent condition in patients with T2DM [22], we can assume that impairments in balance are frequently to be found in patients with T2DM, the latter also developing an increase in the risk of falls. Of a paramount importance for the results of our study is a parallel to a series of other studies which demonstrated that in patients with balance impairment exercise, kinesis and physical therapy significantly improves gait, reaction time and postural stability in these patients [23, 24], thus an early diagnosis followed by a prompt intervention may improve the prognosis in these patients. The possible improvement in the incidence of falls may be of a special importance since it is known that, mainly mediated by hip and wrist fractures, are having a major healthcare cost, for example in 2000 in The United States, the healthcare system spent 19 billion USD on the direct medical costs related to falls injuries, hip fractures alone costing 8.7 billion USD per year [25]. The consecutive reduction of the possible falls, obtained after this kind of interventions is further emphasized by the fact that wound and fracture healing is impaired in patients with T2DM. Regarding the possible treatment approaches for balance problems, it is known that sensibility issues are a frequent cause of these impairments [26] and thus, resolving them a significant positive impact on the balance may be obtained. A feasible approach of this relationship was proposed by Ducic et al. which demonstrated that peripheral nerve decompression leaded to gain of pedal sensibility and consecutive balance impairments in patients with peripheral neuropathy [27].

### Future perspectives

As further perspectives, we aim to develop this cross-sectional study into a prospective one in which we will evaluate the impact kinesiotherapy and physiotherapy in patients diagnosed in the present study that present balance impairment. In addition, we aim to perform standard of care interventions regarding the treatment of DN. All the patients will be followed up and the impact of these combined measures (DN treatment, physiotherapy and kinesiotherapy) will be analyzed. Also a possible further approach will be the evaluation of peripheral microcirculation by transcutaneous oximetry followed by the assessment of the relationship between the already studied components and these newly obtained results. This diagnostic procedure has been widely validated and proved to be a reliable method in this situation [28], all the hypothesis leading to a possible association between neuropathy, balance and impaired peripheral microcirculation.

## Conclusions

The presence of DN in patients with DM is associated with impaired balance and with a consecutively increase in the risk of falls. Patients with DM and DN should have their balance parameters evaluated and if impairments are to be found, in order to decrease the risk of falls, these patients should be included in a rehabilitation program, consisting in physiotherapy and kinesiotherapy, aiming to improve their balance and walking stability.

## References

[1] International Diabetes Federation. IDF Diabetes Atlas, 7 ed Brussels, Belgium: International Diabetes Federation, 2015.

[2] Mota M, Popa SG, Mota E, Mitrea A, Catrinoiu D, Cheta DM, et al Prevalence of diabetes mellitus and prediabetes in the adult Romanian population: PREDATORR study. J Diabetes. 2015 4 7 10.1111/1753-0407.12297 25850521

[3] Lagani V, Koumakis L, Chiarugi F, Lakasing E, Tsamardinos I. A systematic review of predictive risk models for diabetes complications based on large scale clinical studies. J Diabetes Complications. 2013; 27(4): 407–13. 10.1016/j.jdiacomp.2012.11.003 23273850

[4] Giacco F, Brownlee M. Oxidative stress and diabetic complications. Circ Res. 2010; 107(9): 1058–1070. 10.1161/CIRCRESAHA.110.223545 21030723 PMC2996922

[5] Boulton AJ, Malik RA. Diabetic neuropathy. Med Clin North Am. 7 1998; 82(4):909–29. 9706126 10.1016/s0025-7125(05)70029-8

[6] Dyck PJ, Kratz KM, Karnes JL, Litchy WJ, Klein R, Pach JM, et al The prevalence by staged severity of various types of diabetic neuropathy, retinopathy, and nephropathy in a population-based cohort: the Rochester Diabetic Neuropathy Study. Neurology. 1993; 43(4): 817–24. 8469345 10.1212/wnl.43.4.817

[7] Tesfaye S, Selvarajah D, Advances in the epidemiology, pathogenesis and management of diabetic peripheral neuropathy, Diabetes Metab Res Rev. 2012; Suppl 1: 8–14. 10.1002/dmrr.2239 22271716

[8] Tahrani AA, Dubb K, Raymond NT, Begum S, Altaf QA, Sadigi H, et al Cardiac autonomic neuropathy predicts renal function decline in patients with type 2 diabetes: a cohort study. Diabetologia. 2014; 57(6): 1249–56. 10.1007/s00125-014-3211-2 24623102

[9] Karmakar RN, Khandakar MR, Gangopadhyay PK, Ghosh K, Babu AS. Albuminuria and neuropathy in newly detected diabetics: profile and correlation. J Indian Med Assoc. 2011; 109(6): 396–9. 22315767

[10] Dimitropoulos G, Tahrani AA, Stevens MJ. Cardiac autonomic neuropathy in patients with diabetes mellitus. World J Diabetes. 2014; 5(1): 17–39. 10.4239/wjd.v5.i1.17 24567799 PMC3932425

[11] Brown SJ, Handsaker JC, Bowling FL, Boulton AJ, Reeves ND. Diabetic peripheral neuropathy compromises balance during daily activities. Diabetes Care. 2015; 38(6): 1116–22. 10.2337/dc14-1982 25765355

[12] Hewston P, Deshpande N. Falls and Balance Impairments in Older Adults with Type 2 Diabetes: Thinking Beyond Diabetic Peripheral Neuropathy. Can J Diabetes. 2016; 40(1):6–9. 10.1016/j.jcjd.2015.08.005 26778679

[13] Allet L, Armand S, de Bie RA, Golay A, Pataky Z, Aminian K, et al Clinical factors associated with gait alterations in diabetic patients. Diabet Med. 2009; 26(10): 1003–9. 10.1111/j.1464-5491.2009.02811.x 19900232

[14] Herman WH, Pop-Busui R, Braffett BH, Martin CL, Cleary PA, Albers JW, et al Use of the Michigan Neuropathy Screening Instrument as a measure of distal symmetrical peripheral neuropathy in Type 1 diabetes: results from the Diabetes Control and Complications Trial/Epidemiology of Diabetes Interventions and Complications. Diabet Med. 2012; 29(7): 937–44. 10.1111/j.1464-5491.2012.03644.x 22417277 PMC3641573

[15] Muir SW, Berg K, Chesworth B, Klar N, Speechley M. Quantifying the magnitude of risk for balance impairment on falls in community-dwelling older adults: a systematic review and meta-analysis. J Clin Epidemiol. 2010; 63(4): 389–406. 10.1016/j.jclinepi.2009.06.010 19744824

[16] Beauchet O, Fantino B, Allali G, Muir SW, Montero-Odasso M, Annweiler C. Timed Up and Go test and risk of falls in older adults: a systematic review. J Nutr Health Aging. 2011; 15(10):933–8. 22159785 10.1007/s12603-011-0062-0 PMC12878042

[17] Greenberg SA. Analysis of measurement tools of fear of falling for high-risk, community-dwelling older adults. Clin Nurs Res. 2012; 21(1):113–30. 10.1177/1054773811433824 22373731

[18] Manea L, Gilbody S, McMillan D. A diagnostic meta-analysis of the Patient Health Questionnaire-9 (PHQ-9) algorithm scoring method as a screen for depression. Gen Hosp Psychiatry. 2015; 37(1):67–75. 10.1016/j.genhosppsych.2014.09.009 25439733

[19] Pop-Busui R. What do we know and we do not know about cardiovascular autonomic neuropathy in diabetes. J Cardiovasc Transl Res. 2012; 5(4):463–78. 10.1007/s12265-012-9367-6 22644723 PMC3634565

[20] Taguri M, Matsuyama Y, Ohashi Y. Model selection criterion for causal parameters in structural mean models based on a quasi-likelihood. Biometrics. 2014; 70(3):721–30. 10.1111/biom.12165 24621405

[21] Lee IH, Park SY. Impairment of balance in elderly subjects with type 2 diabetes. J Phys Ther Sci. 2014; 26(10):1519–20. 10.1589/jpts.26.1519 25364101 PMC4210386

[22] Vinik AI, Nevoret ML, Casellini C, Parson H. Diabetic neuropathy. Endocrinol Metab Clin North Am. 2013; 42(4):747–87. 10.1016/j.ecl.2013.06.001 24286949

[23] Morrison S, Colberg SR, Parson HK, Vinik AI. Exercise improves gait, reaction time and postural stability in older adults with type 2 diabetes and neuropathy. J Diabetes Complications. 2014; 28(5):715–22. 10.1016/j.jdiacomp.2014.04.007 24929798

[24] Roman de Mettelinge T, Cambier D, Calders P, Van Den Noortgate N, Delbaere K. Understanding the relationship between type 2 diabetes mellitus and falls in older adults: a prospective cohort study. PLoS One. 2013; 8(6):e67055 10.1371/journal.pone.0067055 23825617 PMC3692422

[25] Berry S, Miller R. Falls: epidemiology, pathophysiology and relationship to fracture. Curr Osteoporos Rep. 2008; 6(4):149–154. 19032925 10.1007/s11914-008-0026-4 PMC2793090

[26] Ducic I, Short KW, Dellon AL. Relationship between loss of pedal sensibility, balance and falls in patients with peripheral neuropathy. Ann Plast Surg. 2004; 52(6):535–40. 15166971 10.1097/01.sap.0000122654.65588.f0

[27] Ducic I, Taylor NS, Dellon AL. Relationship between peripheral nerve decompression and gain of pedal sensibility and balance in patients with peripheral neuropathy. Ann Plast Surg. 2006; 56(2):145–50. 16432321 10.1097/01.sap.0000194246.18332.23

[28] Trignano E, Fallico N, Chen HC, Faenza M, Bolognini A, Armenti A, et al Evaluation of peripheral microcirculation improvement of foot after tarsal tunnel release in diabetic patients by transcutaneous oximetry. Microsurgery. 2016; 36(1):37–41. 10.1002/micr.22378 25641727

